# Visualization of antennal lobe glomeruli activated by nonappetitive D-limonene and appetitive 1-octen-3-ol odors via two types of olfactory organs in the blowfly *Phormia regina*

**DOI:** 10.1186/s40851-020-00167-3

**Published:** 2020-11-27

**Authors:** Toru Maeda, Tomoyosi Nisimura, Shunnya Habe, Tatsuya Uebi, Mamiko Ozaki

**Affiliations:** 1 Department of Biology, Graduate School of Science, Kobe University, Nada, Kobe, 657-8501 Japan; 2 Malopolska Centre of Biotechnology, Jagiellonian University, 30-387 Krakow, Poland; 3 College of Bioresource Sciences, Nihon University, Fujisawa, 102-8275 Japan

**Keywords:** Blowfly, Antenna, Maxillary palp, Primary olfactory center, Glomerular mapping, pERK staining, Feeding motivation, Food preference

## Abstract

Appetite or feeding motivation relies significantly on food odors. In the blowfly *Phormia regina*, feeding motivation for sucrose is decreased by the odor of d-limonene but increased by the odor of 1-octen-3-ol odor. These flies have antennal lobes (ALs) consisting of several tens of glomerular pairs as a primary olfactory center in the brain. Odor information from different olfactory organs—specifically, the antennae and maxillary palps—goes to the corresponding glomeruli. To investigate how odors differently affect feeding motivation, we identified the olfactory organs and glomeruli that are activated by nonappetitive and appetitive odors. We first constructed a glomerular map of the antennal lobe in *P. regina*. Anterograde fluorescence labeling of antennal and maxillary afferent nerves, both of which project into the contralateral and ipsilateral ALs, revealed differential staining in glomerular regions. Some of the axonal fiber bundles from the antennae and maxillary palps projected to the subesophageal ganglion (SOG). We visualized the activation of the glomeruli in response to odor stimuli by immunostaining phosphorylated extracellular signal-regulated kinase (pERK). We observed different glomerulus activation under different odor stimulations. Referring to our glomerular map, we determined that antennal exposure to d-limonene odor activated the DA13 glomeruli, while exposure of the maxillary palps to 1-octen-3-ol activated the MxB1 glomeruli. Our results indicated that a nonappetitive odor input from the antennae and an appetitive odor input from the maxillary palps activate different glomeruli in the different regions of ALs in the blowfly *P. regina*. Collectively, our findings suggest that compartmentalization of glomeruli in AL is essential for proper transmission of odor information.

**Supplementary Information:**

The online version contains supplementary material available at 10.1186/s40851-020-00167-3.

## Introduction

Many natural products serve as a means of communication between organisms. For example, some flowering plants provide insect pollinators with attractive sweet nectar in conjunction with floral scents, both of which help their pollinators find and consume food [1], and some have defensive chemicals to prevent herbivores from feeding [2]. Concomitantly, insects have developed adaptive chemosensory systems to select nutritious compounds but avoid noxious substances. Blowflies are known to feed on nectar, and they also serve as pollinators [3, 4]. Studies with *Phormia regina* have long contributed to the understanding of feeding behavior and its neural mechanisms involving the peripheral and central nervous systems [5–17]. Owing to an abundance of early electrophysiological studies with the single sensillar recording method called the tip-recording procedure, the characteristic profile of four functional different gustatory receptor neurons (GRNs) in a gustatory sensillum has been revealed [5, 8, 11, 12, 14]. These GRNs, named for the stimuli to which they respond, are the sugar, salt, water, and bitter taste receptor neurons, respectively. In both *P. regina* and *Drosophila melanogaster*, it is believed that sugar receptor neurons, which can respond to a wide range of nutritious phagostimulants, and bitter taste receptor neurons, which respond to noxious compounds, directly promote positive and negative feeding motivation, respectively [14, 16–21].

On the other hand, olfactory regulation of appetite in insects has not been studied as much as gustatory regulation, despite the wealth of psychological studies of olfactory effects on appetite, feeding preference and other emotional changes in humans and other animals [22–25].

Previously, Maeda et al. [26] categorized 50 types of floral scents into 12 groups according to their nonappetitive, neutral, or appetitive effects on the feeding motivation of *P. regina* after 5 days of dietary experience in the presence or absence of each floral scent. They have also shown that the sensitivity of the proboscis extension reflex (PER) for sucrose feeding was decreased in response to olfactory stimulation with the floral scent of Japanese narcissus, *Narcissus tazetta*, via the antennae, whereas it was increased in response to olfactory stimulation with the floral scent of skunkvine, *Paederia scandens*, via the maxillary palp.

Furthermore, Maeda et al. [27] performed morphological studies to demonstrate the synaptic connections between a portion of the maxillary olfactory receptor neurons (ORNs) and one of the four functionally different GRNs from the single labellar taste sensillum at the subesophageal ganglion (SOG), which is known as the primary gustatory center in the fly brain. Thus, we speculated that an appetitive olfactory input could directly enhance the gustatory response of the sugar receptor neuron through the maxillary ORNs via multiple synaptic connections of those cross-modal receptor neurons in between. Such direct connections between the ORN and GRN may allow the fly to recognize the neural signal of the appetitive odor as a part of phagostimulant taste perception. This mechanism provides a putative explanation for why the odor of 1-octen-3-ol induces appetitive behavior in this fly species. Notwithstanding, the behavioral roles of other maxillary and antennal ORNs have not been characterized by their studies. Maeda et al. [27] suggested that the SOG works as not only the primary gustatory center but also a putative stage of cross-modal connections among gustatory, olfactory, and even mechanoreceptor neurons (MRNs). Nevertheless, clear evidence regarding the innervation of antennal olfactory afferents in the SOG is lacking.

In this study, we constructed a standard glomerular map of the AL in *P. regina* by clarifying the connection of axonal bundles between either maxillary or antennal ORNs and glomeruli with the anterograde staining method. In addition, we also examined which glomeruli are associated with olfactory information processing for nonappetitive and appetitive odors. Based on the newly constructed glomerular map, we identified the glomeruli that individually respond to the nonappetitive and appetitive odors of D-limonene and 1-octen-3-ol, respectively, by staining the phosphorylated form of the extracellular signal-regulated kinase with an anti-pERK antibody [28].

## Materials and methods

### Flies

The blowfly *P. regina* was reared in our laboratory under a 12 L:12 D light-dark cycle at 22 ± 2 °C. The larvae were fed chicken liver and yeast bait (Oriental Yeast Co., Ltd.). Adults were provided with water and 0.1 M sucrose solution in separate cups. Throughout the experiments in this study, we used 4- to 8-day-old adults. The adult flies older than 7 days were nurtured to collect eggs in a separate cage with water, sucrose, and chicken liver. Eggs laid on the chicken livers were collected every morning.

### Anterograde tracing of the antennal and maxillary nerves

**Fig. 1:**
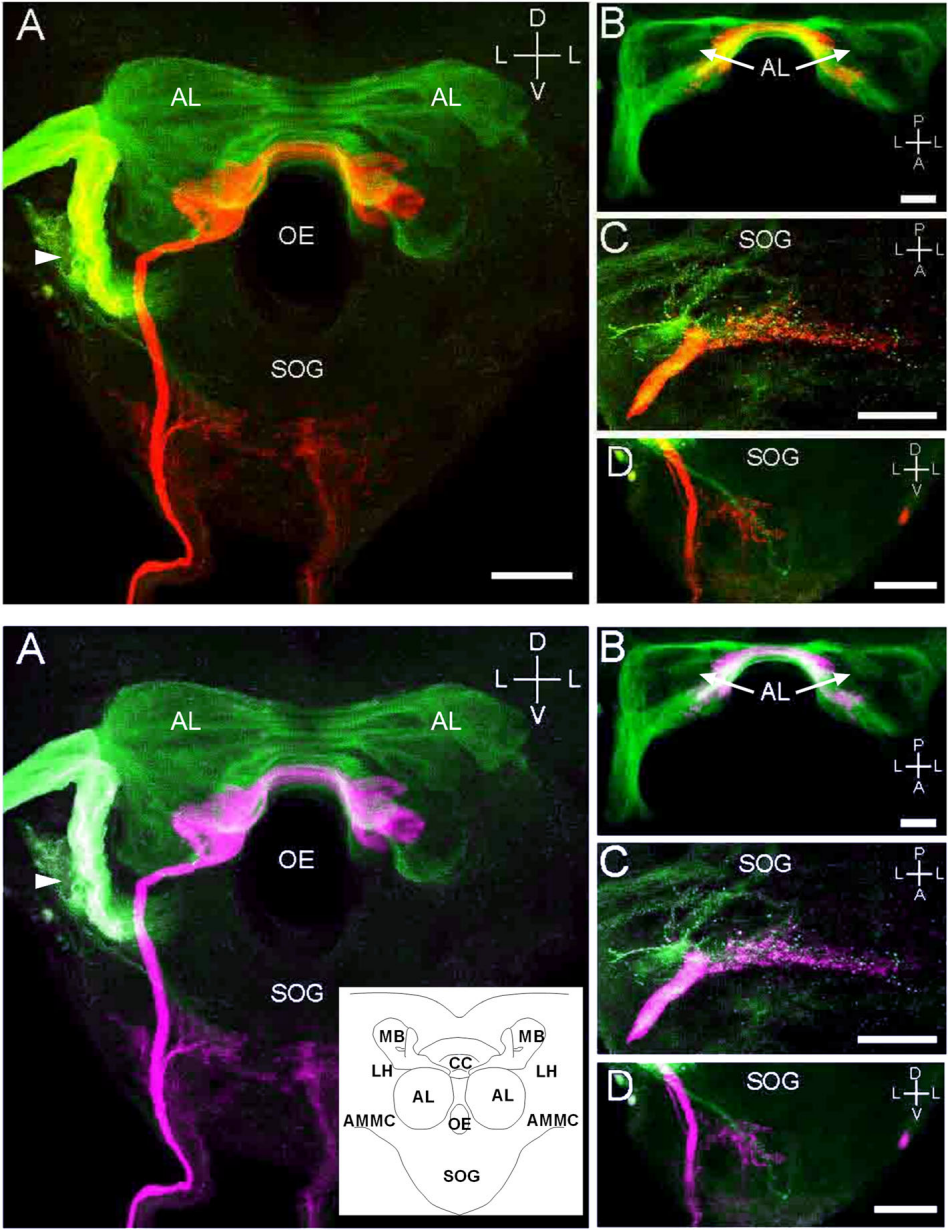
Top: Double labeling of ORNs from the right antenna (green) and the right maxillary palp (red) in the brain. Bottom: Same as Top, but red and green are converted to magenta and cyan, respectively. **a**–**d** are image stacks of 61, 23, 27, and 70 confocal sections at 2.2-, 1.5-, 0.75-, and 0.75-μm intervals, respectively. **a** Frontal view of AL, **b** horizontal view of AL, **c** horizontal view of SOG, and **d** frontal view of SOG. The arrowhead in (**a**) indicates the AMMC region. Bars indicate 50 μm. The inset is a schematic drawing of the neuropil organization of the blowfly brain. CC, central complex; LH, lateral horn; MB, mushroom body; OE, esophagus

**Fig. 2:**
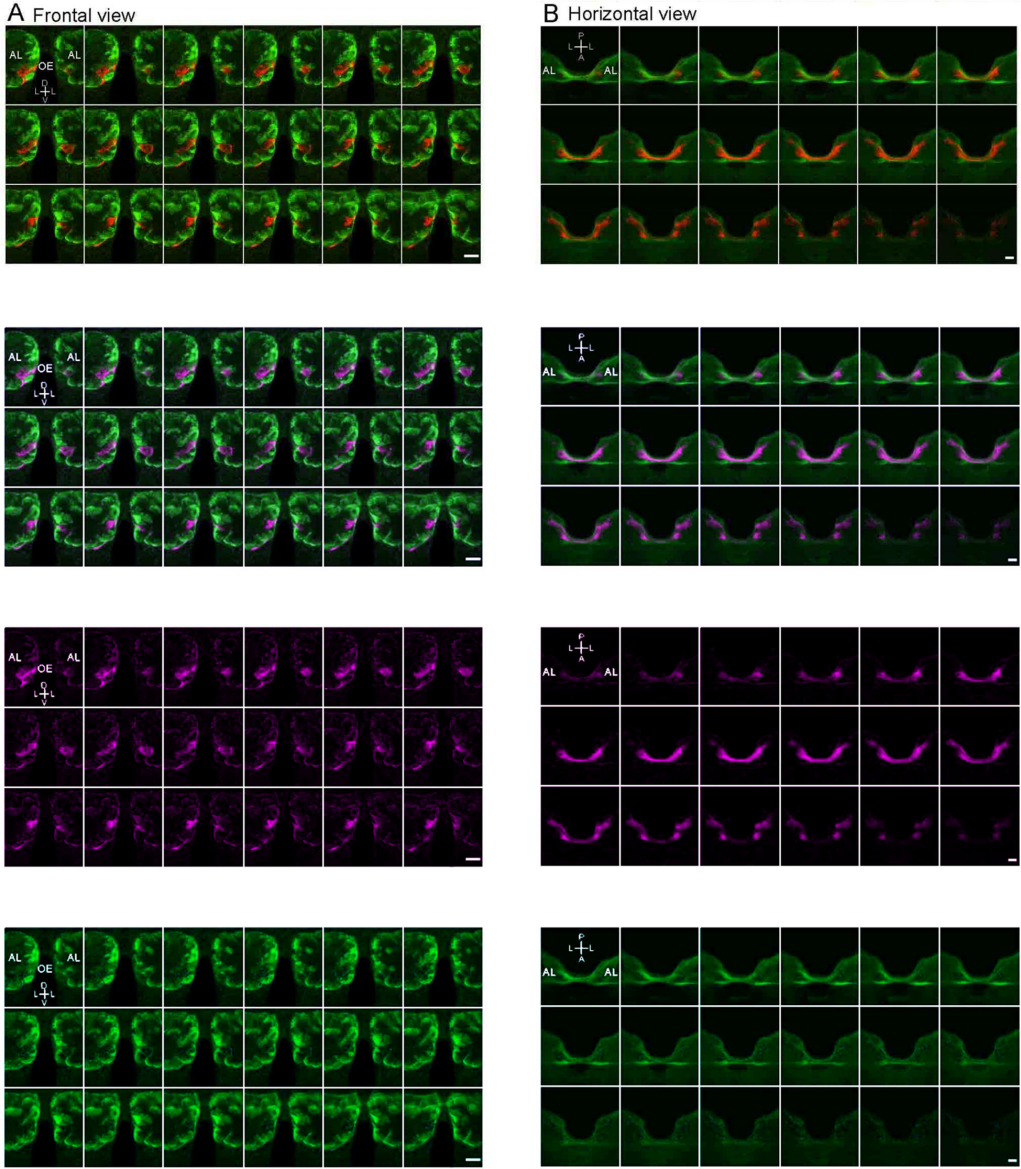
Sequential confocal sections of the AL showing double labeling of ORNs from the right antenna (green or cyan) and the right maxillary palp (red or magenta). **a**–**d** Each plate indicates optical sections from anterior (upper left) to posterior (lower right) at 1.0-μm intervals (range, 24–42 μm in depth from the anterior surface of the lobe) in red-green, magenta-cyan, magenta, and cyan color schemes, respectively. **e**–**h** Each plate represents optical sections from dorsal (upper left) to ventral (lower right) at 0.55-μm intervals in red-green, magenta-cyan, magenta, and cyan color schemes, respectively. Bars indicate 30 μm

**Fig. 3:**
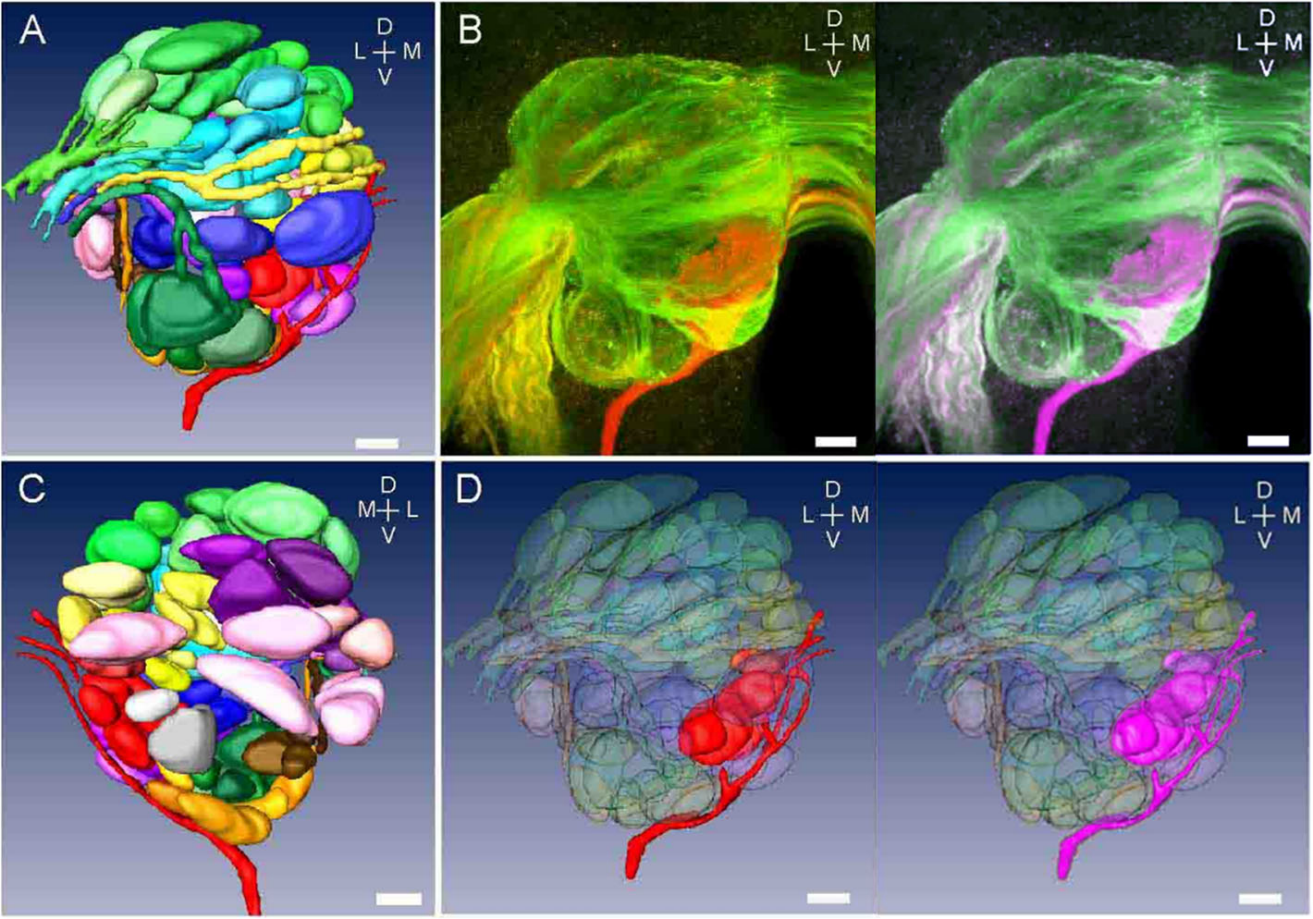
A projection of the confocal images and reconstructed glomerular structure and tracts. **a** and **c**, respectively, show anterior and posterior aspects of the reconstructed right AL. Most glomeruli are categorized into subdivisions using the innervation pattern of the tracts (see Table 1). **b** shows antennal ORNs (green or cyan) and maxillary ORNs (red or magenta) projecting to the right AL. **d** shows glomeruli projecting from the maxillary palps (red or magenta) with transparent images of glomeruli projected from the antennae. Bars indicate 20 μm

Adult females 6 to 8 days after emergence were immobilized by cooling in a plastic dish (38 mm diameter, 14 mm height) set on ice. For double staining of axonal projections from antennae and maxillary palps, the right antenna was cut by hand with microscissors at the level of the second flagellar proximal segment, and the right maxillary palp was cut at the proximal end. Small crystals of lysine-fixable dextran fluorescein (D-3306, Molecular Probes, Inc., Eugene, OR, USA) were immediately placed onto the lesion of the right antenna, and lysine-fixable dextran tetramethylrhodamine (D-3308, Molecular probes, Inc.) was placed onto the lesion of the right maxillary palp. Afterward, the flies were kept for 1 to 2 days at room temperature. The brains were dissected and then fixed overnight at room temperature in 1% paraformaldehyde in 18.4 mM ZnC_l2_, 135 mM NaCl, and 25 mM glucose [27]. After fixation, the specimens were washed with phosphate-buffered saline (PBS, pH 7.4); serially dehydrated with 70, 80, 90, 95, 100, and 100% ethanol for 5 min per solution; and then permeated with methyl salicylate. Some specimens of the fixed brain were washed with PBS, embedded in 5% agarose (A-0169, Sigma, St. Louis, MO, USA), and sectioned horizontally at a thickness of 100 μm with a Microslicer (DTK-1000, Dosaka EM Co.). They were dehydrated and mounted in NEW MX (Matsunami Glass Ind., Ltd.). Those whole-mount or sliced specimens were observed under a confocal laser scanning microscope (LSM 510, Carl Zeiss). For color presentation in Figs. 1, 2 and 3, we show those figures not only in real color but also in pseudocolor, with red and green replaced by magenta and cyan, respectively.

### Three-dimensional computer analysis

Whole-mount brain specimens were imaged with a confocal laser scanning microscope using a Plan-Neofluar 40× objective (numerical aperture, 0.75). Serial optical slices were acquired from posterior to anterior at approximately 1.0-μm intervals. For the reconstruction of glomeruli, we selected 12 scanned preparations with good staining, and a confocal stack of approximately 60 optical sections was analyzed using Amira software (Indeed Visual Concepts GmbH, Berlin, Germany; TGS Inc.). In each optical section, we demarcated each glomerulus and antennal and maxillary nerve and drew the contours by hand using a Wacom LCD tablet (PL550, Wacom Co., Ltd.).

### PER test for assessing appetite

In the present study, the term “appetite” is used to indicate feeding motivation. According to Nisimura et al. [15], we evaluated appetite as the reciprocal of the threshold concentration of sucrose necessary to elicit the PER conducted as a prerequisite for feeding. High and low PER thresholds to sucrose indicate decreased and increased appetite in individual flies, respectively. Odor sources were prepared in different concentrations in silicon oil (KF-96-10cs; Shin-Etsu Chemical Co., Ltd., Tokyo, Japan) and examined for the PER test. We counted the number of flies exhibiting PER to various concentrations of sucrose in the absence (control condition) or presence of d-limonene or 1-octen-3-ol odor (test condition). Sucrose-dose-dependent PER curves were constructed and compared between the control and test conditions. We performed the PER experiment with 5 sets of 20 flies randomly selected from a group of flies that simultaneously hatched from a single egg mass, i.e., a total of 100 flies were used for a PER experiment as either control or test individuals. Those flies were immobilized by securing the wings with aluminum clothes pegs. Before the PER test, either the antennae or the maxillary palps were cut with microscissors to determine which olfactory organ is involved in the PER threshold increase or decrease. At the beginning of the PER test, flies were provided distilled water to satiation. Six concentrations of sucrose solutions were prepared by fourfold serial dilution with distilled water starting from 1 M (2^0^, 2^− 2^, 2^− 4^, 2^− 6^, 2^− 8^, and 2^− 10^ M). The labellar chemosensilla were carefully stimulated with each concentration of sucrose to prevent the fly from ingesting the stimulus solution. The stimulation began with the lowest concentration of sucrose. The PER test was carried out in the absence and then the presence of an odor source (5 μl of pure d-limonene or 1-octen-3-ol on a cotton swab) that was set 2 cm away from the head of a tested fly. The experiment with 20 flies each was repeated five times under the same odor condition, and the percentage of flies showing PER was recorded in 100 flies in total. Using the Wilcoxon signed-rank test, we compared the PER threshold in each individual between the control (with no odor) and test conditions with d-limonene or 1-octen-3-ol odor, as shown in [14]. Although the Steel-Dwass test was used in the cited paper, we used the Wilcoxon signed-rank test for the corresponding data in this study.

### Immunohistochemical detection of activated glomeruli using anti-pERK antibody

#### Anti-pERK antibody

The anti-pERK antibody specifically binds phosphorylated extracellular signal-regulated kinase (pERK). We used phospho-p44/42 MAPK (Erk1/2) (Thr202/Tyr204) (D13.14.4E) XP rabbit mAb #4370 (Cell Signaling Technology) to identify activated glomeruli in the fly brain. This antibody has been used in similar studies in zebrafish [28].

#### Exposure of flies to stimuli

The flies were fixed by securing their wings with aluminum clothes pegs. The labella with gustatory sensilla were ablated because the GRN for bitter taste can respond to d-limonene vapor [8, 14], and either the antennae or maxillary palps were also cut off. After 1 h, we set the odor source (5 μl of pure d-limonene or 1-octen-3-ol on a cotton swab) 2 cm away from the tested fly and let it smell the odor for 10 s via either the preserved antennae or maxillary palps. The fly was immediately anesthetized on ice for approximately 5 min. The head was cut off, and the remaining olfactory organs were removed to prevent further stimulation with background odor, such as the odor from the fixative solution. The isolated head was then fixed in 4% paraformaldehyde at room temperature overnight.

#### Tissue sample preparation

On the next day, the brain was dissected in fly Ringer’s solution under a stereomicroscope (SZX9 Olympus) and then immediately immersed in 10-fold-diluted Histo VT ONE (Nacalai Tesque, Inc.) at 90 °C for 45 min for activation. After cooling to room temperature, the brain was washed three times with fly Ringer’s solution containing 0.1% Triton-X, immersed in Blocking One (Nacalai Tesque, Inc.) for 20 min and washed three times with fly Ringer’s solution containing 0.1% Triton-X. The brain was incubated at room temperature overnight in the primary antibody solution containing anti-pERK antibody (1:500 dilution) in 95% Can Get Signal Immunostain Solution B (Toyobo Inc.) and 5% Blocking One. The brain was subsequently washed three times with fly Ringer’s solution containing 0.1% Triton-X and incubated in the dark at 4 °C overnight in a solution of Alexa Fluor 594 goat anti-rabbit IgG (Molecular Probes) containing 95% Can Get Signal Immunostain Solution B and 5% Blocking One. The brain was washed three times with fly Ringer’s solution containing 0.1% Triton-X and three times with plain fly Ringer’s solution. The brain was dehydrated by incubation in an ethanol series (70, 80, 90, 95, 100, and 100%) for 5 min each and penetrated with methyl salicylate. Finally, the brain sample was placed on a glass plate with a small amount of methyl salicylate and observed under a confocal laser microscope (FV1000 Olympus Co.).

## Results

### Sensory projections from antennae and maxillary Palps

Staining of either the antennal nerve with dextran fluorescein (green) or the maxillary nerve with dextran tetramethylrhodamine (red) revealed that the afferents innervated most of the antennal lobe glomeruli in the ipsilateral and contralateral hemibrains (Fig. 1a and b), even when the fluorescent dyes were introduced only from the right side. Furthermore, many fibers from the antennae bypassed the lobe ventrolaterally and terminated in the lateral deutocerebrum, which, in all likelihood, contained the antennal mechanosensory and motor center (AMMC). This area of the putative AMMC (arrowhead in Fig. 1a), into which MRNs may project from the Johnston organ, was intensely labeled in all 23 specimens tested. Fibers were found to project from the antennae into the SOG in 17 of the 23 specimens (Fig. 1a). In the SOG, the terminal areas of the antennal afferents were near those from the maxillary afferents in 10 of the 23 specimens (Fig. 1c and d).

A distinct fiber bundle of maxillary ORNs was found to project into the SOG through the labial nerve, as previously reported by Maeda et al. [27], and further ascended into the ipsilateral and contralateral ALs (Fig. 1). This red projection pattern was observed in all specimens tested. The labeled terminals have typical glomerular-type structures and innervate a group of glomeruli via the ventromedial edge of the AL (Fig. 1a and b). If the axonal route on either side of the maxillary palp follows mirror symmetry, as was suggested by Maeda et al. [27], each glomerulus should receive innervation from both maxillary palps. In Fig. 1a, some red labeling is also seen from the left labial nerve to the SOG. From the characteristic staining pattern [26], this staining may be an artifact caused by the labellar GRNs that were injured due to technical difficulties in the delicate surgery to ablate the maxillary palps. If this happened, some fluorescent dye might accidentally enter the GRNs.

To confirm that afferents from antennae and maxillary palps separately project into specific glomeruli from each other, we precisely analyzed each of six best-stained optical sections among all 19 brain specimens and three of the four horizontally sectioned specimens. The red fluorescence dye from the antenna and the green fluorescence dye from the maxillary palp did not overlap at all, indicating that the afferent nerves from the antennae and maxillary palps project into completely different glomeruli in the AL (Fig. 2).

### Three-dimensional reconstruction of the antennal lobe

Figure 3 shows the surface reconstruction of glomeruli and a trace of the right side AL in a female fly. In the frontal view, the labeled glomerular mass of the right AL is ovary shaped (125.2 ± 15.4 μm (mean ± SD) in width along the median-lateral axis and 141.0 ± 21.6 μm in height along the dorsoventral axis) (Fig. 3a). Figure 3d shows a cleared antennal nerve and its terminal glomeruli so that a maximal nerve and its terminal glomeruli are seen in red or magenta. The representative tracts from an identified antennal nerve and a maxillary nerve (see Table 1) were reconstructed (Fig. 3a and c). In every fly, each side of the AL had 80 glomeruli, 73 and 7 of which were innervated by antennal and maxillary ORNs, respectively (Table 1). Specifically, the antennal nerve splits into 10 tracts at the entrance of the AL, and they enter the AL in a posteromedial direction. Each tract innervates 3–14 glomeruli in the ipsilateral AL. Six of them (DA, IAa, IAb, IAc, IAd, and VA in Table 1) innervate glomeruli in the anterior part of the AL. Another four tracts (DP, IP, VPa, and VPb in Table 1) run through the posterolateral side of the outer layer of the lobe and innervate glomeruli in the posterior part of the AL. A tract from the maxillary nerve (Mx in Table 1) runs through the SOG in a dorsal direction and innervates the AL. This tract enters the AL on the ventral side and runs along with the outer layer of the AL in a dorsomedial direction, ultimately innervates the glomeruli located in the ventromedial region of the AL.

**Table 1:** Subdivision of 80 glomeruli of *P. regina*

Nerve tract area in AL glomerular region (Abbreviation)		Color in Figs. 3 and 4	Number of glomeruli
Nerve	Vertical axis – Craniocaudal axis		
Antennal nerve	Dorso – Anterior (DA)	Green	14
	Intermediate – Anterior a (IAa)	Light blue	8
	Intermediate – Anterior b (IAb)	Yellow	11
	Intermediate – Anterior c (IAc)	Purple	5
	Intermediate – Anterior d (IAd)	Blue	5
	Ventro – Anterior (VA)	Dark green	6
	Dorso – Posterior (DP)	Dark purple	5
	Intermediate – Posterior (IP)	Pink	7
	Ventro – Posterior a (VPa)	Orange	5
	Ventro – Posterior b (VPb)	Brown	3
	Unidentified (UN)	Gray	4
Maxillary nerve	Dorso – Anterior (MxA)	Red	3
	Ventro – Anterior (MxB)	Red	2
	Intermediate – Posterior (MxC)	Red	2

**Fig. 4:**
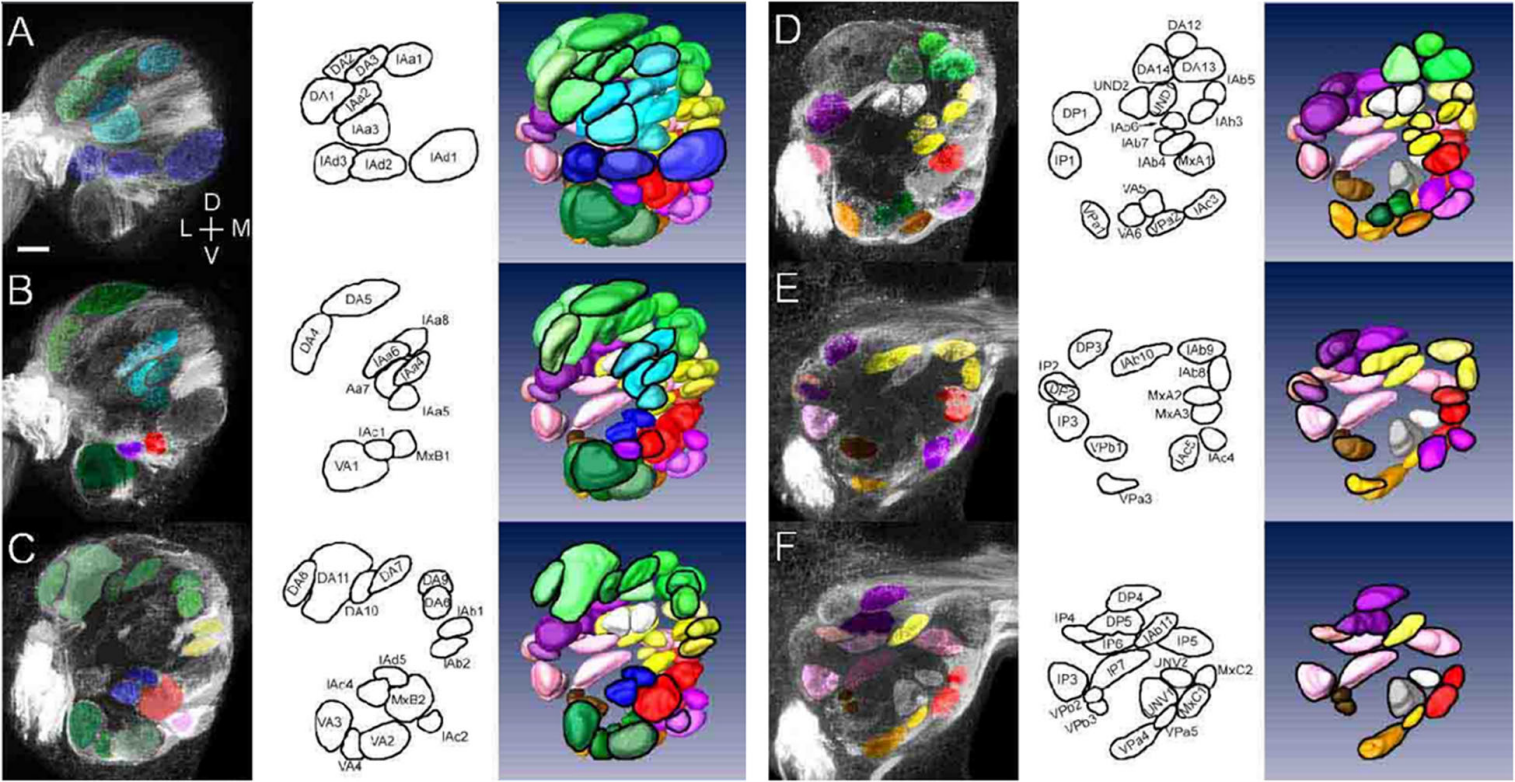
Representative confocal stacks and partial three-dimensional reconstructions of the right AL. Six frontal planes through the AL from anterior to posterior (**a**–**f**) are shown. The color coding is identical to that of the subgroups of glomeruli (see Fig. 3 and Table 1). Left column: Each plane is a representative stack of 10 confocal images at 0.9-μm intervals (stack size, 9 μm). Colors are superimposed on the glomeruli identified in each confocal stack. Middle column: Identified and demarcated glomeruli (black outline). Right column: Partial three-dimensional reconstructions. The top panel **a** shows the whole pattern. The subsequent panels **b**–**f** show the structures of the inner glomeruli. The outlined glomeruli are the ones identified in each confocal stack in the left column. Bar indicates 20 μm

The antennal lobe glomeruli can be subdivided into 11 groups based on their innervation by the tracts mentioned above. The DA tract enters the AL on the most dorsal side and innervates 14 glomeruli located in the dorsal region of the AL (green in Fig. 3a and c, Fig. 4a–d). Between the most dorsal and ventral tracts (DA and VA), four anterior tracts (IAa, IAb, IAc, and IAd) enter the AL. The IAa tract runs more dorsally than the other three tracts and innervates eight glomeruli located in the anterior region of the AL (light blue in Fig. 3a, Fig. 4a and b). The IAb tract runs beneath the IAa tract and passes anteriorly across the AL in a medial direction (yellow in Fig. 3a and c, Fig. 4a–f). Eleven glomeruli innervated by this tract were observed in the medial part of the AL, with nine in the posterior side of the AL (IAb3–IAb11). The IAc and IAd tracts run close together at the AL entrance, although they innervate different groups of glomeruli from each other. Of the five glomeruli innervated by the IAc tract, one was observed in the anteroventral side of the lobe, and the other four were observed in the medial-ventral region of the lobe (purple in Fig. 3c, Fig. 4a–f). The IAd tract innervates five anterior glomeruli, three of which are large and positioned medially (blue in Fig. 3a, Fig. 4a–c).

### Receptor organs for olfactory inputs that affect appetite

**Fig. 5:**
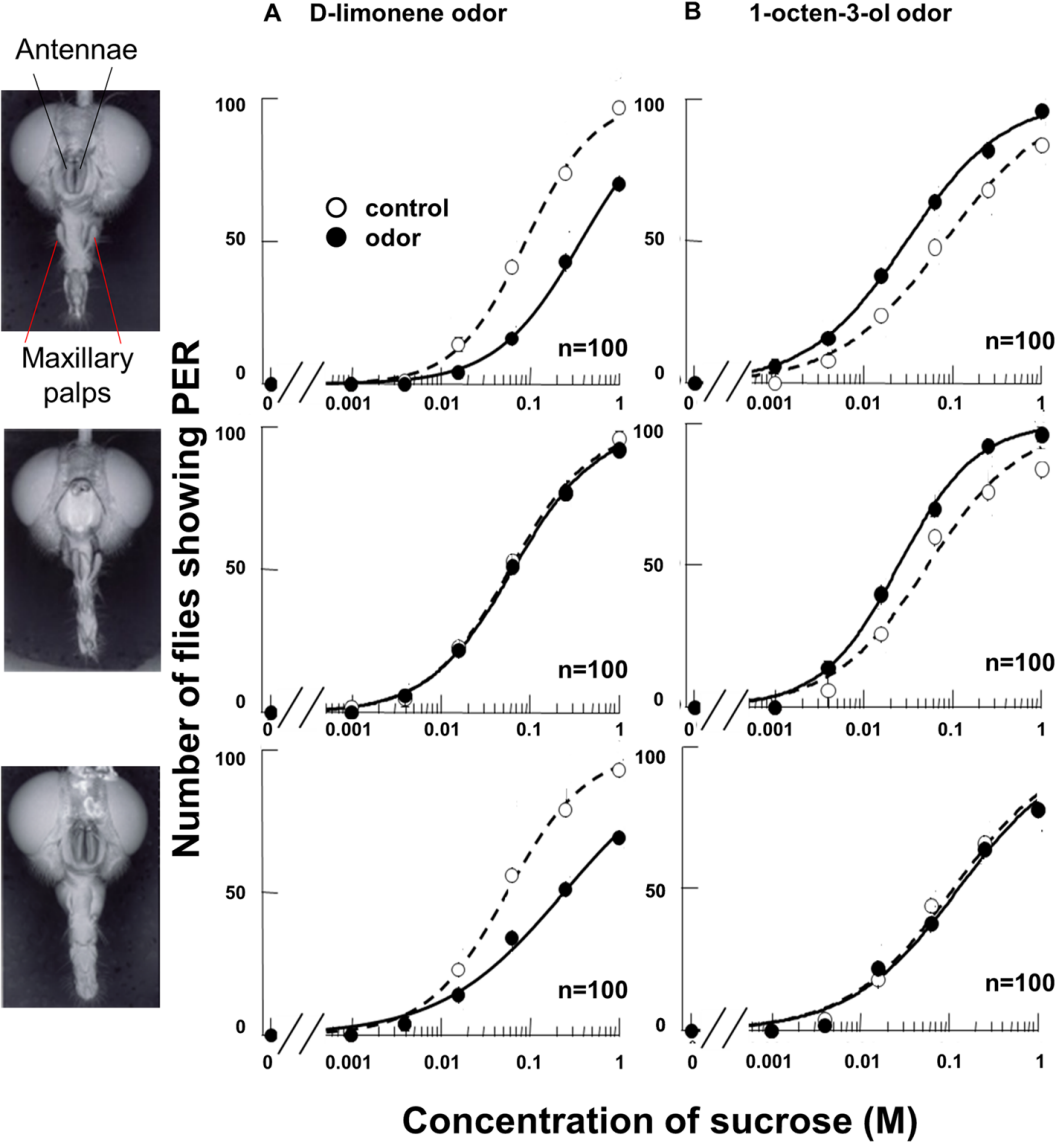
Effect of d-limonene or 1-octen-3-ol odor on the PER in olfactory organ-ablated flies. Left panels show three different fly preparations having intact maxillary palps (MP) and antennae (AN) (top), intact maxillary palps but no antennae (middle), and intact antennae but no maxillary palps (bottom). **a** Sucrose concentration–PER curves in the absence (open circles) and presence (closed circles) of d-limonene odor. **b** Sucrose concentration–PER curves in the absence (open circles) and presence (closed circles) of 1-octen-3-ol odor. The number of flies exhibiting a PER is plotted against sucrose concentration; the curves of best fit were constructed using a curve-fitting program, Igor (HULINKS Ltd.). There was a significant difference between the PER thresholds in the two curves (Wilcoxon signed-rank test, *p* < 0.05; *n* = 100)

Flies have two types of olfactory organs: the antennae and the maxillary palps. Thus, we determined which olfactory organ is responsible for the decrease or increase in PER sensitivity against sucrose by using a nonappetitive odor of d-limonene and appetitive odor of 1-octene-3-ol. The PER sensitivity depends on the dose of the odorant, and the threshold concentrations of d-limonene and 1-octene-3-ol odors to change the PER sensitivity against 125 mM sucrose were 1:1000 and 1:100 dilutions, respectively (Supplementary Fig. 1). In intact or maxillary palp-ablated flies, d-limonene odor decreased the PER threshold to sucrose solutions (Wilcoxon signed-rank test, *P* < 0.05, *n* = 100, Fig. 5a Top and Bottom). However, the d-limonene odor did not affect the PER to sucrose in antenna-ablated flies (Fig. 5a Middle). The result indicates that the antennae, but not the maxillary palps, mediate the suppression of PER caused by d-limonene.

In contrast, we found that the maxillary palp is responsible for the enhancement of PER to sucrose. In intact or antennae-ablated flies, 1-octen-3-ol odor increased the PER threshold to sucrose solutions (Wilcoxon signed-rank test, *P* < 0.05, *n* = 100, Fig. 5b Top and Middle). However, the 1-octen-3-ol odor did not affect the PER response of maxillary palp-ablated flies to sucrose (Fig. 5b Bottom). Therefore, the PER to sucrose in flies is reduced when they sense d-limonene with the antennae and enhanced when they sense 1-octen-3-ol with the maxillary palps.

### AL glomeruli activated by olfactory inputs that affect appetite

**Fig. 6:**
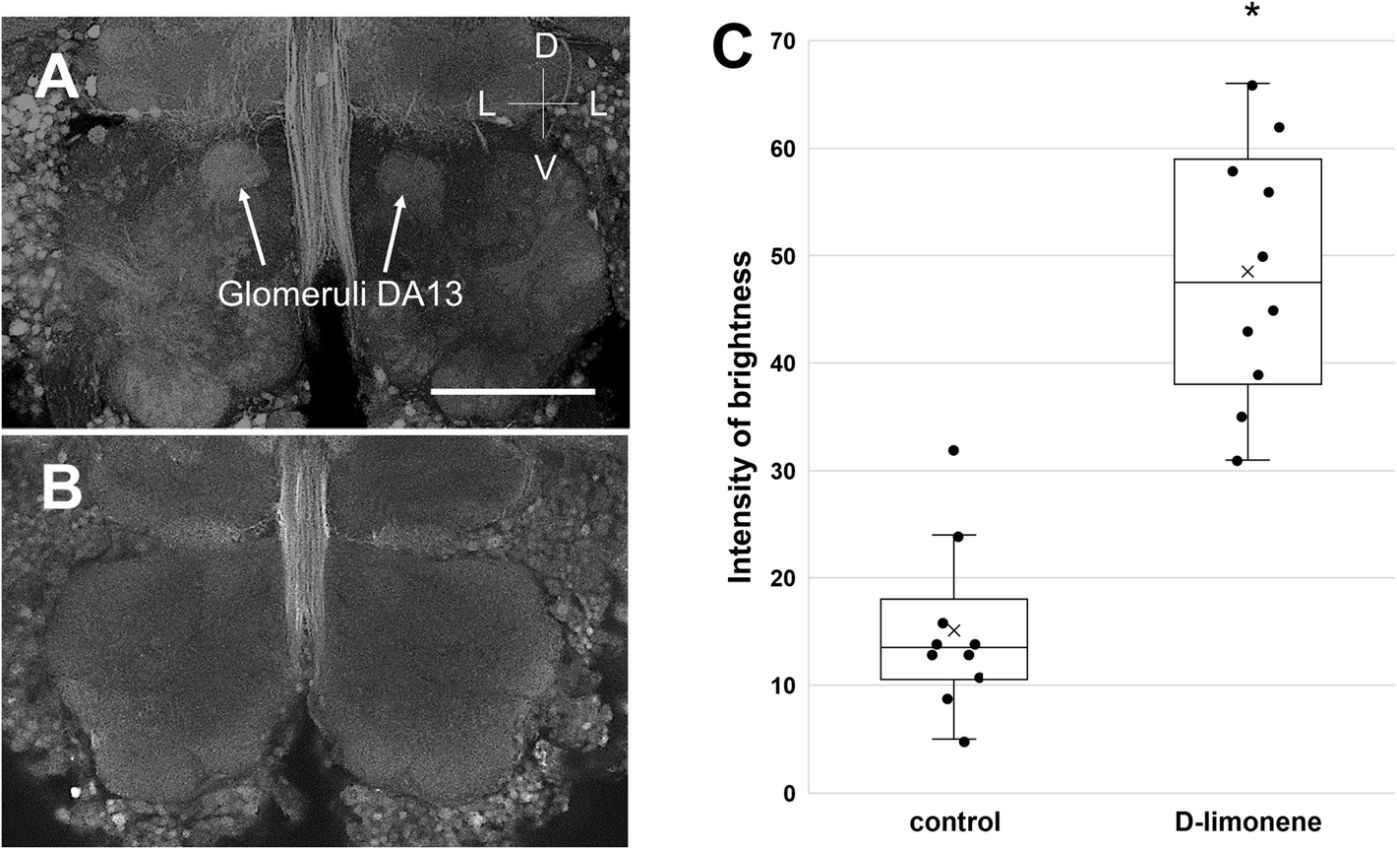
AL glomeruli activated by D-limonene odor stimulation via maxillary palps. **a** A pair of glomeruli DA13 labeled by anti-pERK immunostaining. **b** Control image without odor stimulation. The bar indicates 100 μm. **c** Boxplot comparing the brightness of activated glomeruli DA13 in the test (*n* = 10) with the background brightness in the control (*n* = 10). Boxplot whiskers are 1.5× interquartile range

**Fig. 7:**
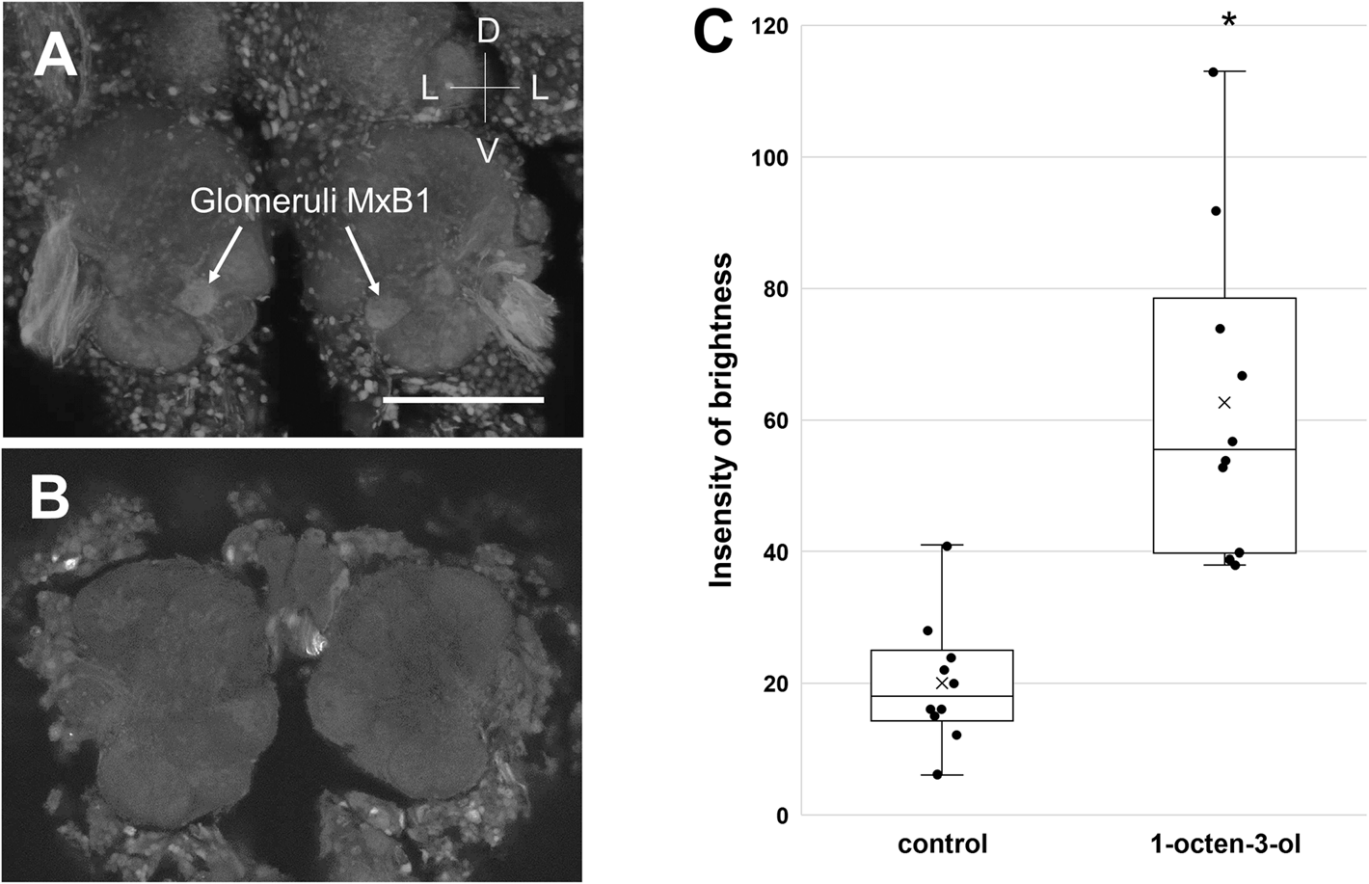
AL glomeruli activated by 1-octen-3-ol odor stimulation via maxillary palps **a** A pair of glomeruli MxB1 labeled by anti-pERK antibody staining. **b** Control image without odor stimulation. The bar indicates 100 μm. **c** Boxplot comparing the brightness of activated glomeruli MxB1 in the test (*n* = 10) with the background brightness in the control (*n* = 10). Boxplot whiskers are 1.5× interquartile range

Figure 6a shows a representative image of activated glomeruli, where the pERK accumulated after the stimulation of antennae with d-limonene odor. In 10 of the 12 independent samples, we observed the activation of a specific pair of glomeruli symmetrically located on either side of the ALs (Fig. 6a, Supplementary Fig. 2A). No significant signals of pERK were observed in any of the 23 samples in the control experiment with no odor stimulation, except for the midline of the brain (Fig. 6b). We identified the activated pair of glomeruli as DA13 at the dorsomedial side of the ALs by referring to the glomerular map (see Fig. 4d). In contrast, we found significant activation of the specific glomeruli, namely, MxB1, at the ventromedial side of the ALs in 10 of 11 independent samples after stimulation of the maxillary palps with a 1-octen-3-ol odor (Fig. 7a, Supplementary Fig. 2B). No glomerular activation was observed in the control experiment (Fig. 7b). We quantitatively evaluated activity in 10 pairs of glomeruli and compared the control and test by measuring brightness using ImageJ (Figs. 6c and 7c). We subtracted the background signals on each micrograph of the specimen and measured the median brightness of the active glomerulus in the test and the median background brightness of the same part on the brain in the control. There were statistically significant differences in brightness between the control and test groups (Mann-Whitney’s U test, *P* < 0.05, *n* = 10).

## Discussion

We constructed a glomerular map of the AL in *P. regina* based on imaging of olfactory neurons visualized by anterograde staining. The result is useful for identifyingspecific glomeruli that are activated by nonappetitive or appetitive odor in the brain of the blowfly.

Anterograde fluorescence labeling of antennal and maxillary afferent nerves revealed differential staining between the glomeruli that project into the contralateral and ipsilateral ALs. Some of the axonal fiber bundles from the antennae and maxillary palps projected differently into the SOG. Visualization of activated glomeruli in the AL revealed that stimulation of the antenna with d-limonene activated the DA13 glomeruli, whereas stimulation of the maxillary palp with 1-octen-3-ol activated the MxB1 glomeruli. In the present paper, we mainly examined olfactory activation in the antennal glomeruli. However, this histochemical method with anti-pERK antibody will be useful to stain other regions in the olfactory circuit.

### Functionally different olfactory inputs and roles of the antennae and maxillary Palps

Maeda et al. [27] have already reported that the odor of 1-octen-3-ol, when perceived by the maxillary palps but not by the antennae, increases the appetite of *P. regina* for sucrose. Here, we further demonstrate that the odor of d-limonene, which is known to be fatal toxic when orally administered [14], suppressed the appetite for sucrose only via the antennae and not via the maxillary palps (Fig. 5a). Previously, although Nisimura et al. [15] examined the olfactory effect of d-limonene on the appetite of the blowfly, it was still unclear which organ—the antenna or the maxillary palp—is responsible for appetite regulation. Because the maxillary palps are located near the labellar taste organs on the proboscis, they are closely exposed to food odors during the extension of the proboscis for feeding. Thus, we suspected, at least in this fly species, that increased appetite in response to certain odors mediated via the maxillary palps might enhance the intake of nutritious foods [27].

Previous morphological data indicated that some of the maxillary ORNs project into the AL, while others project into the SOG [29]. In a subsequent study, Maeda et al. [27] showed that the maxillary ORNs connect to the single labellar GRN via synapses in the SOG. These data suggest that appetitive odor inputs from the maxillary palp can positively modify feeding behavior by enhancing the phagostimulant response of the sugar receptor neuron. Our results may suggest that some antennal ORNs also project into the SOG (Fig. 1c and d). Otherwise, some GRNs or MRNs of gustatory or mechanosensilla, if any are present on the antennae, might project to the SOG. The functional role of these neurons and the biological significance of their projection to the SOG are still unknown. Although it has scarcely been investigated, olfactory information might also go to the SOG via some afferent nerves from the antennae (see Fig. 1c and d), which may integrate with a different modality of information conveyance. The resting afferents from either the maxillary palps or the antennae reach the AL, and then the olfactory information may further transfer to the higher brain via projection neurons for various processing or modification. Evidence suggests that the SOG is neuropil, wherein various neural signals for different sensory modalities can be concentrated and integrated [27].

### AL glomerular map and compartmentation

The AL is the primary olfactory center in insects, analogous to the olfactory bulbs (OBs) in vertebrates [30]. Homologous neuropils in insects and vertebrates consist of multiple glomeruli. In *D. melanogaster*, precise glomerular mapping has revealed that each olfactory sensillum houses two to four ORNs, for which the distribution, sensitivity, and expression of olfactory receptor (*Or*) genes have been reported [31–39]. The maxillary and antennal ORNs project to distinct glomerular areas within the AL. Of the 43 glomeruli per AL in *D. melanogaster*, six are projected from the maxillary ORNs [40–44]. Glomerular organization in the AL has been precisely reported in several insect species [45–47]. Except for orthoptera [48], one ORN terminates in one glomerulus. Neural projection from the maxillary ORNs to the AL glomeruli was first determined in *Drosophila* [29] and then mosquito [49]. In the blowfly, Maeda et al. [27] found that the maxillary ORNs terminate not only in both the ipsilateral and contralateral ALs but also in the ipsilateral SOG. In the fruit fly and mosquito*,* the antennal and maxillary ORNs have been reported to project to both the ipsilateral and contralateral ALs [50, 51]. However, this is not always true of insects in general; there are many insect species that have olfactory projections only to the ipsilateral AL. Hence, Diptera might have a special olfactory information perception system, although its biological meaning is unknown. We show here that *P. regina* has 80 glomeruli per AL, of which seven project from the maxillary ORNs (Table 1). These seven maxillary-palp-innervated glomeruli are located together in the dorsomedial area of the AL, which is different from the area consisting of glomeruli innervated by the antennal afferents (Fig. 3). These two glomerular areas are further divided based on neural tracts (Figs. 3 and 4). Thus, the total compartmentation of the AL in *P. regina* constitutes 14 glomerular groups, each consisting of 2 to 14 glomeruli (Table 1). The separation of glomeruli into discrete compartments is sometimes responsible for the pattern recognition of odors with a standard biological function. For example, in the Japanese carpenter ant, a specific compartment known as T6 consists of more than 100 glomeruli that are innervated by the cuticular hydrocarbon (CHC)-sensitive sensilla, which allows the recognition of a variety of CHC patterns of mixed compositions [52], thus enabling worker ants to discriminate non-nestmates from nestmates. In mice, a specific compartment located in the OB consists of the dorsal domain of glomeruli and is involved in the olfactory recognition of predators such as foxes and cats [53]. Considering the chemical defense system using isothiocyanates, which has evolved especially in Brassica plants [2], we examined the odor of homogenized seedlings of *Arabidopsis* instead of d-limonene or noxious odor. We obtained the pERK staining data, as shown in Supplementary Fig. 3.

### Nonappetitive and appetitive odor-dependent activation of glomeruli via different olfactory receptor organs

Both qualitative and quantitative associations involving olfactory stimulation, AL activity, and olfactory preference have been reported [54, 55]. However, few studies have shown the pattern of glomerular activation in the AL during the response to odors that have significant psychological or behavioral effects. In the present study, we successfully identified a pair of DA13 glomeruli activated by nonappetitive d-limonene odor in an anterodorsal compartment consisting of 14 glomeruli (Fig. 6). This observation suggests that *P. regina* expresses an *Or* gene essential for d-limonene odor recognition in a specific antennal ORN. Thus, the expression of *Or* genes in some proper ORNs is adaptive for flies to avoid noxious substances with oral toxicity, such as d-limonene [14], although it is unknown how often flies encounter d-limonene or d-limonene-containing noxious compounds in nature. We identified another pair of glomeruli, MxB1, in the anterodorsal region, which were activated upon stimulation of the antenna-ablated fly with an appetitive odor of 1-octen-3-ol (Fig. 7). Thus, the compartments in which DA13 and MxB1 reside are distinctively separated from each other.

## Conclusions

Referring to the newly constructed glomerular map in *P. regina*, we identified DA13 as the glomeruli activated by a nonappetitive odor input of d-limonene via antennae and MxB1 as the glomeruli activated by an appetitive odor input of 1-octen-3-ol via maxillary palp. Our results suggest that compartmentalization in AL regions is required for nonappetitive and appetitive odor information transmission in *P. regina*. However, it remains unclear how odor information is integrated and reflected in fly feeding behavior; this question could be addressed by studies of higher brain regions. We found that two different nonappetitive odorants activate glomeruli in the same DA region (see Supplementary Fig. 3).

In general, food odor preference can frequently change over the course of a lifetime due to altering dietary habits. For example, such a change in food odor preference occurs in *P. regina* [15, 26]. Higher brain mechanisms involving hormonal regulation may be concerned, but further activation patterns in the AL might change by dietary experience with some odors. Our experimental design will be useful for addressing the remaining questions concerning appetite modulation by odors.

## Supplementary Information

**Additional file 1:** **Fig. S1.** Effect of d-limonene or 1-octen-3-ol odor on PER sensitivity at different dilutions. Suppression of PER sensitivity by d-limonene odor (A) and enhancement of this reflex by 1-octen-3-ol (B). The PER test at 125 mM sucrose was conducted with 20 flies per dilution rate of odorants, which were serially diluted 10-fold with silicon oil, and the percentage of flies showing a PER is plotted against the dilution rate of the odorant (mean ± SEM; *n* = 5). Compared to an odor-free control, significant differences (asterisks) were found at a 1:1000 dilution of d-limonene and at a 1:100 dilution of 1-octen-3-ol (Dunnett’s test, *p* < 0.05; *n* = 5).

**Additional file 2:** **Fig. S2.** AL glomeruli activated by d-limonene odor stimulation via antennae (A) and 1-octen-3-ol odor stimulation via maxillary palps (B). The bar indicates 100 μm. Arrows in A indicate glomeruli DA13, as shown in Fig. 6, and arrows in B indicate glomeruli MxB1, as shown in Fig. 7.

**Additional file 3: Fig. S3**. AL glomeruli activated by stimulation with the odor of *Arabidopsis thaliana*. The *Arabidopsis* seedlings were homogenized and left at room temperature for 30 min to generate volatile isothiocyanates, whose odor has a repellent effect against animals. After the exposure of flies to the odor, the same histochemical staining as in Figs. 6 and 7 was conducted with an anti-pERK antibody. (A): A representative image of fly glomeruli stimulated by the odor of *Arabidopsis* seedling homogenate. (B): An image of a control experiment with no odor stimulation. (C): Boxplot comparing the brightness of activated glomeruli DA9 in the test (*n* = 5) with the background brightness in the control (*n* = 5). The bar indicates 100 μm. Boxplot whiskers are 1.5× interquartile range.

## Data Availability

The datasets used and/or analyzed during the current study are available from the corresponding author on reasonable request.

## Abbreviations

AL(s): Antennal lobe(s)
AMMC: Antennal mechanosensory and motor center
A-P: Anterior-posterior
D-V: Dorsal-ventral
GRN(s): Gustatory receptor neuron(s)
I: Intermediate
L-M: Lateral-medial
OB: Olfactory bulb
*Or*: Olfactory receptor gene
ORN(s): Olfactory receptor neuron(s)
PER: Proboscis extension reflex
pERK: phosphorylated extracellular signal-regulated kinase
SD: Standard deviation
SOG: Subesophageal ganglion
