# Supplementary figures and images for: Visualization of antennal lobe glomeruli activated by nonappetitive D-limonene and appetitive 1-octen-3-ol odors via two types of olfactory organs in the blowfly Phormia regina
Source: Zoological Lett. 2020 Nov 27;6:16. doi: 10.1186/s40851-020-00167-3 (PMC7694429; doi:10.1186/s40851-020-00167-3)

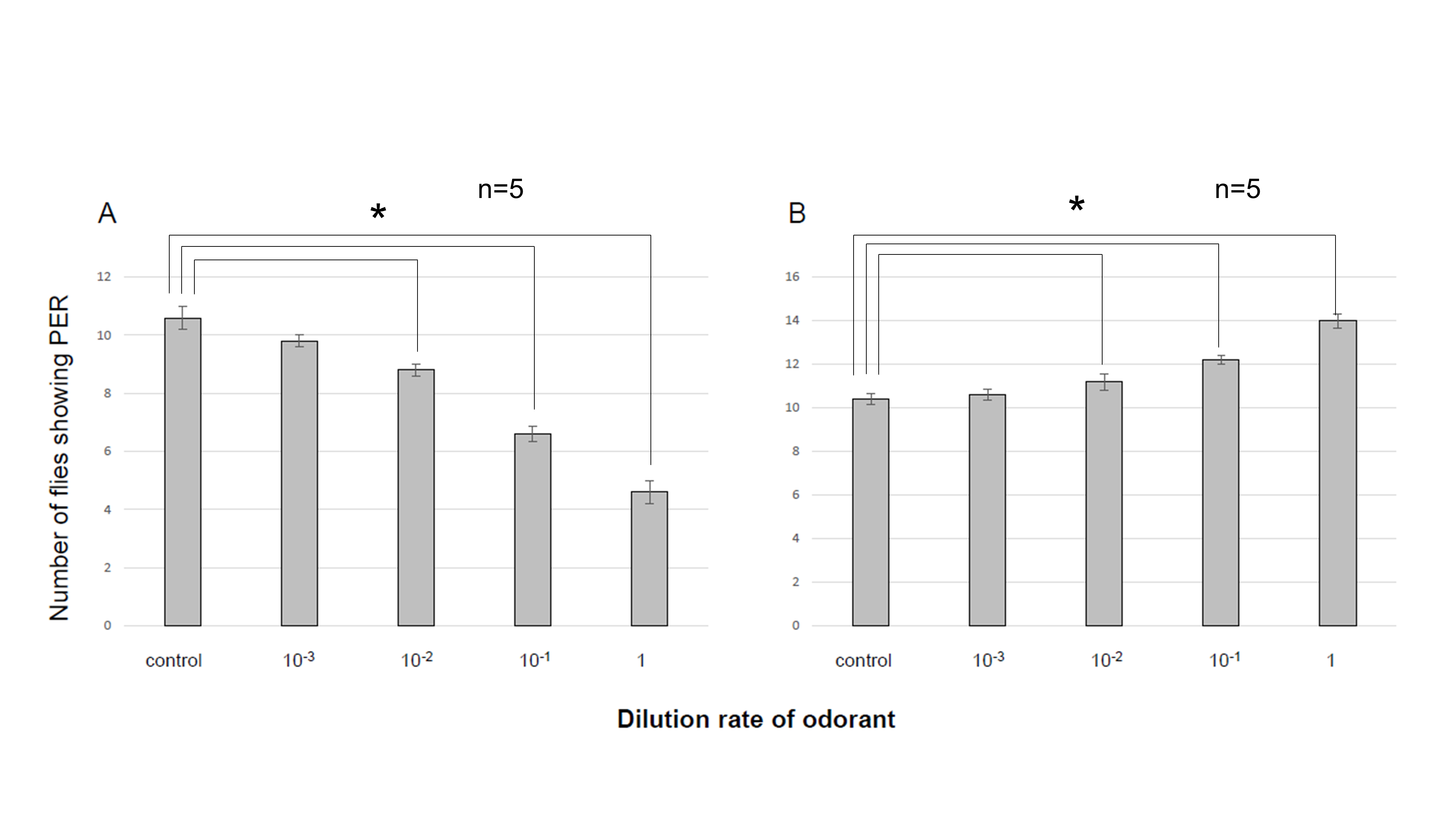

Supplement: Supplementary file 1 — Additional file 1: Fig. S1. Effect of d-limonene or 1-octen-3-ol odor on PER sensitivity at different dilutions. Suppression of PER sensitivity by d-limonene odor (A) and enhancement of this reflex by 1-octen-3-ol (B). The PER test at 125 mM sucrose was conducted with 20 flies per dilution rate of odorants, which were serially diluted 10-fold with silicon oil, and the percentage of flies showing a PER is plotted against the dilution rate of the odorant (mean ± SEM; n = 5). Compared to an odor-free control, significant differences (asterisks) were found at a 1:1000 dilution of d-limonene and at a 1:100 dilution of 1-octen-3-ol (Dunnett’s test, p < 0.05; n = 5). [file 40851_2020_167_MOESM1_ESM.tif]

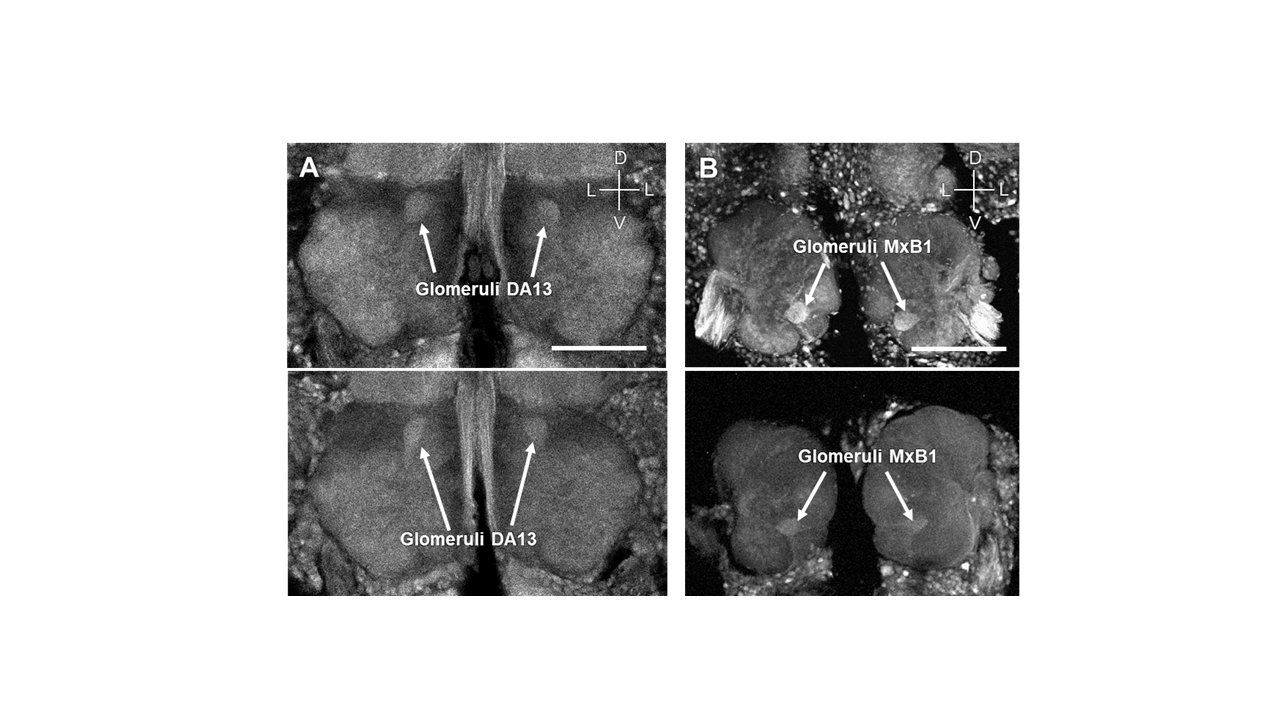

Supplement: Supplementary file 2 — Additional file 2: Fig. S2. AL glomeruli activated by d-limonene odor stimulation via antennae (A) and 1-octen-3-ol odor stimulation via maxillary palps (B). The bar indicates 100 μm. Arrows in A indicate glomeruli DA13, as shown in Fig. 6, and arrows in B indicate glomeruli MxB1, as shown in Fig. 7. [file 40851_2020_167_MOESM2_ESM.tif]

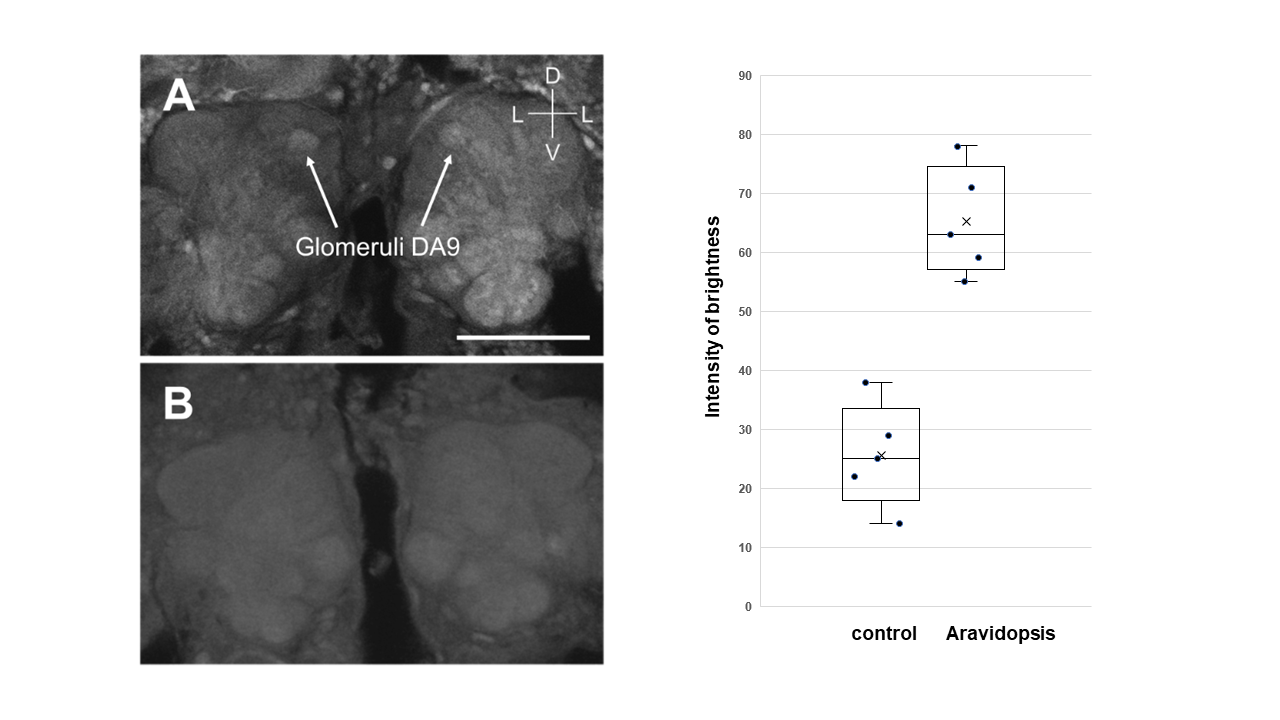

Supplement: Supplementary file 3 — Additional file 3: Fig. S3. AL glomeruli activated by stimulation with the odor of Arabidopsis thaliana. The Arabidopsis seedlings were homogenized and left at room temperature for 30 min to generate volatile isothiocyanates, whose odor has a repellent effect against animals. After the exposure of flies to the odor, the same histochemical staining as in Figs. 6 and 7 was conducted with an anti-pERK antibody. (A): A representative image of fly glomeruli stimulated by the odor of Arabidopsis seedling homogenate. (B): An image of a control experiment with no odor stimulation. (C): Boxplot comparing the brightness of activated glomeruli DA9 in the test (n = 5) with the background brightness in the control (n = 5). The bar indicates 100 μm. Boxplot whiskers are 1.5× interquartile range. [file 40851_2020_167_MOESM3_ESM.tif]
